# Resignation of the Faculty

**Published:** 1865

**Authors:** 


### RESIGNATION OF THE FACULTY.

At the close of the last session of the Ohio College of Dental Surgery, the entire Faculty tendered their resignations to the board of trustees, which was accepted.

This was done, not because there was a want of harmony or unanimity in the Faculty, for the utmost good feeling has prevailed, but the Faculty took the view, that as there was to be a reorganization of the board of trustees, and their election placed in the hands of the college association, that a reorganization of the Faculty would be desirable, presuming it would be much to the advantage of the school, and that in no event could it operate an injury. Some of the Faculty decline reappointment, and all would have preferred to work for the college in some other way. Two of the new Faculty have occupied chairs in the institution before, viz: Drs. Watt and H. R. Smith. Of their ability nothing need be said here; the profession understand that. Dr. Kearnes, Prof. of Anatomy, has been a very successful teacher a number of years. Dr. Spalding, Prof. of Institutes of Dental Science, is eminently fitted for his position; a man of extensive scientific and professional attainments, he will bring to his chair a prestige not to be excelled. A slight modification has been made in the chair, physiology is in connection with anatomy; metallurgy with mechanical dentistry; therapeutics with chemistry; and dental hygiene with operative dentistry.

The Faculty will be assisted by three demonstrators, one in anatomy, one in operative dentistry, and one in mechanical dentistry. It is proposed under this management to make the course of instruction as thorough as it is possible in the present status of the profession.

Dr. James Taylor is Emeritus Professor of Institutes of Dental Science.

